# Archaeological evidence of resource utilisation of walrus, *Odobenus rosmarus*, over the past two millennia: A systematic review protocol

**DOI:** 10.12688/openreseurope.17197.1

**Published:** 2024-04-24

**Authors:** Danielle L. Buss, Katrien Dierickx, Mohsen Falahati-Anbaran, Deirdre Elliot, Lisa K. Rankin, Peter Whitridge, Brenna Frasier, Jean-Simon Richard, Youri van den Hurk, James H. Barrett

**Affiliations:** 1 Norges teknisk-naturvitenskapelige universitet Vitenskapsmuseet, Trondheim, Trøndelag, 7491, Norway; 2 Memorial University of Newfoundland, St. John's, Newfoundland and Labrador, Canada; 3 Nova Scotia Museum, Halifax, Nova Scotia, Canada; 4 Musee des Îles de la Madeleine, Les Îles de la Madeleine, Quebec, Canada

**Keywords:** Walrus; zooarchaeology; historical ecology; NISP; hunting; systematic review protocol

## Abstract

The walrus, *Odobenus rosmarus,* is an iconic pinniped and predominant molluscivore that is well adapted to Arctic and subarctic environments. Its circumpolar distribution, large body size and ivory tusks facilitated its vital role as food, raw material (for tools and art), income, and cultural influence on many Arctic Indigenous communities for millennia. Intensification of hunting (often due to the arrival of Europeans, especially between the 16 ^th^ and 19 ^th^ centuries) to obtain ivory, hide, blubber and meat, resulted in diminished, sometimes extirpated, walrus populations. Zooarchaeological, artefactual and documentary evidence of walrus material has been collated at local and regional scales and is frequently focused on a specific culture or period of time. Systematic collation of this evidence across the Northern Hemisphere will provide insight into the chronology and circumpolar distribution of walrus hunting and provide a tool to document societal change in walrus resource use. Here, we lay out a systematic review protocol to collate records of archaeological walrus artefacts, tusks and bones that have been documented primarily within published literature to archive when and where (as feasible) walrus extractions occurred between 1 CE and 2000 CE. These data will be openly available for the scientific community. The resulting dataset will be the first to provide spatiotemporal information (including the recognition of knowledge gaps) regarding past walrus populations and extirpations on a circumpolar scale. Our protocol is published to ensure reproducibility and comparability in the future, and to encourage the adoption of systematic review methodology (including pre-published protocols) in archaeology.

## 1. Background

Coastal ecosystems are often highly productive and biodiverse as frequent upwellings, terrestrial coastal nutrient inputs, variable freshwater inputs, and tidal flows encourage high levels of primary productivity ^1– 5^. Consequently, around a billion humans inhabit coastal regions and are dependent on the oceans for food ^6, 7^. Combined, human affinity to inhabit coastal regions and population expansion has resulted in approximately 85% of global coastlines being anthropogenically altered ^8^. Thus, coastal marine species have been exploited, sometimes unsustainably, for prolonged periods of time, relative to offshore species, resulting in extinctions or extirpations of some coastal animal populations ^9– 15^.

The walrus ( *Odobenus rosmarus)* is a predominantly coastal Arctic and subarctic species that has been hunted for millennia for food, hide, and ivory, with significant declines between the 16 ^th^ and 18 ^th^ centuries; in some localities, potential declines are evident by the 13th to 14th centuries ^11, 16– 22^. Walruses play a keystone role in local food webs, feeding predominantly on molluscs, with occasional predation on fish and smaller pinnipeds ^23– 29^. They are heavily reliant on sea ice for resting, feeding and nursing young ^30– 33^. Moreover, as a keystone species they have a disproportionate impact on their environment and cohabiting species. For example, by creating gaps in the sea ice with their tusks (for breathing holes and to access food), they modify the habitat by increasing the amount of light and oxygen that enters the nearby water column, often creating pockets of increased productivity. Further, they control prey abundance through predation effects, and translocate nutrients through movement and defecation, again contributing to primary productivity ^31, 33– 35^. As walruses and ice cover are intrinsically linked, good documentation on the past distribution of this taxon is also vital in order to understand both the response of northern ecosystems to global change (cf. 36) and potential temporal changes in the behaviour of walrus vis-a-vis sea ice ^35, 37, 38^. Further information will help to understand how northern hemisphere (predominantly Arctic and subarctic) ecosystems and human societies have previously varied in response to environmental and cultural pressures.

Walruses are culturally invaluable to many Indigenous communities ^18, 35, 37, 39– 42^. For example, in areas where walruses occur, the cultural identities of Indigenous Arctic communities among the Inuit, Yup’ik and Chukchi, strongly align with walrus. In these areas, walrus have been hunted for centuries as part of a traditional way of life in order to obtain food, hide, blubber and materials for tools ^18, 21, 35, 37, 43^. Traditional hunting techniques include the use of umiaqs (large skin boats), kayaks and harpoons in order to obtain food, hide, blubber and materials for tools. The continued survival of the walrus is very important for these communities.

In the North Atlantic, early evidence of subsistence hunting of walrus occurs in Maritime Archaic assemblages, from ca. 5500-1500 BCE, and at sites representing the Early Pre-Dorset or Independence I cultures in Eastern Arctic ca. 2500 - 2000 BCE, but with substantial evidence increasing since 500 BCE ^44– 48^. In addition to subsistence hunts, walruses have also been targeted for economic purposes. Ivory tusks are highly prized and have been popular for trade in Eurasia from at least the 9 ^th^ c. CE onwards, mostly by European settlers and/or seasonal hunters in Iceland, the Barents Sea region and (from the late 10th century) Greenland ^49^. More intensive (sometimes industrial) walrus hunting for tusks, oil and hide following European colonisation resulted in diminished and sometimes completely extirpated walrus populations, such as those from Iceland (already in the Viking Age), the Canadian Maritimes (by the late 18th century) and Svalbard (prior to their protection since 1952) ^11, 17, 22, 50– 53^. In the North Pacific, Indigenous communities such as the Chukcki, Inuit, Yup’ik, and their ancestors, have hunted walrus for over a millennium primarily for food, hides and ivory ^21, 54^; with several communities still hunting North Pacific walrus ( *Odobenus rosmarus divergens*) for subsistence today ^54^.

Evidence of past human predation on walrus has been documented using counts of faunal remains of walrus at archaeological sites, predominantly at local and occasionally regional scales (e.g. 39, 55– 57). Ethnographic evidence and written documentation surrounding hunting events have also been documented ^20, 53, 58^. Zooarchaeological finds of walrus from sites typically consist of fragmentary bone and tooth/tusk remains and/or worked ivory artefacts ^59– 61^. The correlation between the number of identified zooarchaeological walrus specimens (NISP) and socioeconomic shifts in past societies is evident. Early Dorset sites show an increase in active walrus exploitation related to changes in technology and social organisation relative to Pre-Dorset sites ^46^. In contrast, Late Dorset sites show a decrease in walrus remains relative to Early Dorset sites, attributed to reduced sea ice extent resulting in decreased accessibility to walrus for coastal communities that did not extensively use watercraft ^62^.

Arctic and subarctic ecosystems are currently undergoing a period of rapid environmental change ^63, 64^, including, warmer temperatures, reduced seasonal sea ice cover ^65– 69^, species compositional changes ^70– 72^ and increased occurrences of harmful algal blooms ^73, 74^. Over the Holocene, northern hemisphere habitats have undergone both cooler and warmer periods, particularly affecting regions with seasonal ice cover like Arctic and subarctic environments, potentially influencing walrus populations ^75– 78^. Reductions in sea ice platforms are already forcing walrus populations to haul-out on land, and they are now frequently observed congregating in large groups ^38, 79^. Such sea ice reductions will increasingly require individuals to swim longer distances to visit current feeding sites which is energetically costly and may result in changes to foraging site usage ^35, 38, 80, 81^. A good understanding of how walruses have historically responded to environmental change may help to predict how they will respond to the ongoing climatic change of the 21 ^st^ century.

### 1.1 Objective

Using a systematic review protocol, we will summarise the archaeological evidence of human utilisation of walruses between 1 CE and 2000 CE across the Northern Hemisphere. The time periods between 1 CE and the present day were characterised by large-scale environmental, demographic and societal change (e.g., 21, 82– 95) and during this time, significant expansion occurred within Arctic and subarctic Indigenous communities ^90, 96– 98^. In addition, there were increased interactions with European migrants and seafarers benefitting from ocean resources, including walrus, via expanding trade networks ^99– 104^. Additionally, walruses predominantly inhabit Arctic and subarctic habitats that are known to have been characterised by cooler and warmer periods throughout the Holocene, likely impacting walrus distribution and availability ^32, 81, 105, 106^. Thus, systematically documenting spatiotemporal variations in the occurrence and size of walrus extractions during this time will provide insight into associations between historic resource utilisation, societal and environmental change.

## 2 Methods

### 2.1 Data type

In order to identify spatiotemporal variation in human utilisation and distribution of walrus populations over the past 2000 years, and how these relate to societal and environmental change, three datasets will be compiled from published literature using predefined search terms (see Section 2.3): (i) faunal count data from archaeological reports - recorded as the number of identified walrus specimens (NISP); (ii) reports of artefacts manufactured from walrus tissues; and (iii) faunal count data of four ice-obligate pinniped species in order to infer past ice-cover and climate. Additionally, information on the introduction and occurrence of walrus hunting practices, alongside written records of walrus hunting events will be recorded ad hoc, in order to contextualise the archaeological evidence; however, a thorough review of historical evidence is beyond the scope of this study.

*(i) Faunal counts of walrus specimens*

Following 107, faunal count data documented in the form of the number of identified specimens (NISP, see 108) will be collated from the archaeological literature. Faunal remains, such as bones, cheek teeth, and tusks, are typically identified by researchers with good anatomical experience (e.g., zooarchaeologists) and recorded in terms of NISP within an archaeological assemblage (e.g. 109). Additionally, the presence or absence of walrus remains, even for sites without NISP data, will be documented for all relevant archaeological assemblages within the published literature, dated between 1 CE - 2000 CE, situated in coastal regions (< 30 miles inland), and located between latitudes 40 and 90 degrees north, including the known historical latitudinal distribution of walruses.

*(ii) Reports of walrus artefacts*

Artefacts discovered during archaeological investigations, or otherwise curated as heritage objects that have known archaeological provenance, are often reported separately from zooarchaeological material. Individual objects or counts of artefacts of the same material and/or ‘type’ (e.g. chess piece) are often reported (e.g. 110, 111). Various artefacts were manufactured from walrus carcasses, including (but not limited to): (i) harpoon heads, support pieces and handles produced from tusk and tooth ivory (e.g. 112, 113); (ii) art and symbolic objects, including decorative reliefs and sculptures of varying size and complexity, produced from tusk and cheek tooth ivory and/or worked bone (e.g. 111, 114, 115); (iii) wall and roof coverings, skin boat coverings, ropes, thongs, bags, belts, bicycle seats, and wallets made out of tanned walrus hides ^19^; and (iv) gaming pieces, dentures, umbrella handles, whistles and other small objects fashioned from the ivory of walrus tusks ^19^. Artefacts made from walrus remains can occur over a much larger spatial area than faunal remains because these items are more likely to be distributed through trade networks ^22, 49, 50^. Although oil extracted from walrus blubber was incorporated into margarine, train oil and soap ^116^, and walrus meat eaten or used as animal feed, evidence of these in the archaeological record is limited due to their relative lack of preservation. We will document evidence of walrus artefacts ad hoc whilst conducting zooarchaeological literature searches. Objects will be excluded if evident from the available source that their collection history breaks the 1970 UNESCO convention on trade of illicit antiquities.

*(iii) Faunal counts of four ice-obligate pinniped species*

To infer likely environmental correlates associated with the occurrence of walrus faunal remains in coastal regions, zooarchaeological NISP data of four ice-obligate pinnipeds (bearded seal, *Erignathus barbatus;* ringed seal, *Pusa hispida;* harp seal, *Pagophilus groenlandicus;* hooded seal *, Cystophora cristata*) will also be collated. NISP data will be collated from within the results of our literature searches for walrus (rather than independent searches for each species) and used to infer change in ice-free and ice-covered regions over time.

### 2.2 Scoping searches

Published literature containing NISP data of walrus at archaeological sites were identified using scoping searches in August 2023 and the search terms *Walrus OR * *Odobenus rosmarus* AND *NISP OR *Archaeology. Search terms were inputted into Web of Science (WoS), Scopus and Google Scholar, independently and the number of records from each platform compared (see Supplementary Table S1). This was repeated using 22 non-english languages resulting in a total of approximately 39,000 and 2,900 records using Google Scholar (Aug 2023) and JSTOR (Jan 2024, english search also included on this date), respectively (see Supplementary Table S2). Twelve of the first 20 unique publications on Google Scholar using the terms Walrus* AND Zooarchaeology* provided archaeological faunal evidence of walrus, with a broad range of time periods spanning 4500 BP to the 19th century. This scoping search suggests an initial percentage of 60% of publications containing informative data using these search terms; although we note that the number of pertinent hits will vary, especially as more closely matching hits will appear earlier in the search list.

### 2.3 Literature search protocol

To obtain the data summarised in Section 2.1, Web of Science (WoS), Scopus, JSTOR and Google Scholar will be systematically searched for relevant sources using the search terms above. Additionally, one research platform, JSTOR, will be searched for academic publications using the non-English search terms shown in Supplementary Table S2, with pilot searches finding ~2,900 hits across the 22 languages. Google Scholar will not be used as it is beyond the scope of this study to include all 39,000 non-english searches. Moreover a substantial number of hits seem unrelated, primarily arising from surname matches. Titles, abstracts, and key words of the identified studies will be reviewed to assess whether they are likely to contain information on walrus faunal remains between 1 CE and 2000 CE. Additional relevant studies will be identified using the R Package CitationChaser ^117^ and backward and forward chasing of accepted studies (see 2.4 below for criteria). One researcher will perform literature searching, screen titles and abstracts of potential studies, and extract data into a pre-designed relational database; 5% of studies will be randomly screened and re-extracted by a second researcher.

### 2.4 Study inclusion criteria

Studies will only be included if they meet the following criteria:

1. They contain information on walrus faunal remains or artefacts either as presence/absence or in the form of the raw number of identified specimens (NISP) with associated chronological information dated between 1 CE and 2000 CE.
2. NISP or artefact data must relate to the species, *Odobenus rosmarus* (under one of the following english or non-english search terms ‘walrus’ or ‘tusked seal’ or ‘morse’ or ‘sea oxen’ or ‘ *Odobenus rosmarus*’ or *‘Odobenus*’, or ‘ *Odobaenus’* or ‘海象’ or ‘바다 코끼리’ or ‘anjing laut’ or ‘الفظ’ or ‘Морж’ or ‘hvalross’ or ‘hvalros’ or ‘morša’ or ‘rostungur’ or ‘valross’ or ‘mursu’ or ‘Aaveq’ or ‘Iuġuaq’ or ‘yuġġuaq’ or ‘aivik’ or ‘Aiviq’ or ‘ᐊᐃᕕᖅ’ or ‘Ayveq’ or ‘Aivuk’ or ‘Asveq’ or ‘amgadaq’ or ‘morsa’ or ‘セイウチ’; see Table S2 for associated languages (further language inclusion was beyond the scope of this study).
3. The associated chronological precision is ≤500 years.
4. Geolocations for each site can be identified from reported site information within a minimum precision of 1.0 decimal degrees.

### 2.5 Data extraction and management

All studies that meet eligibility criteria, are accessible, and within the limits of copyright will be downloaded, PDFs and citations imported into a reference manager and duplicates removed. Data will be manually added from individual reports into a custom-designed OpenOffice 4.1.13 database ^118^ following ^107^ but with some exceptions (see Supplementary Table S3.). More specifically the following details will be extracted for each study:

***2.5.1. Publication details.*** Publication title, subsection title (if applicable, like subchapter, chapter or article), publication year, type of publication specifying publisher details for books or the journal name, author names, corresponding author’s name and email, DOI (if available; otherwise provide the URL for published web-based studies), intellectual property status, and acknowledgement of external source of additional metadata, such as georeferenced information.

***2.5.2. Archaeological assemblage details.*** An archaeological assemblage is a defined stratigraphic or spatial component (or subcomponent) of an archaeological site. For each archaeological assemblage containing identified walrus fauna, the following detailed information will be recorded: the decimal latitude and longitude of the archaeological site (when available); the name of the assemblage, the country, province/state/or equivalent (e.g. county), the site type (e.g. shipwreck, refuse pit), the archaeological site name and number or code (if available), and the modern settlement name (as applicable). Furthermore, the date of the assemblage exactly as stated in the original study, alongside the start and end dates (in CE - CE format), and information on the method used to determine chronology will also be recorded.

***2.5.3. Taxon and faunal data.*** In reports where faunal presence or counts of walrus are identified, the associated information will be recorded: the species name as recorded in the original study and the total number of identified specimens (NISP) within a given assemblage; ‘ *presence*’ will be recorded for assemblages with known walrus fauna or artefacts but without available count data. Where applicable, the total number of identified and unidentified specimens in the assemblage and which taxonomic groups this includes (only walrus; all pinnipeds; all mammals; or all vertebrates) will also be recorded. As taxonomic certainty can vary depending on the experience of the zooarchaeological research ^119– 121^, when available the name of the analyst who performed the faunal analysis will be reported, as will the method used for faunal recovery. For reports where walrus NISP data are available alongside other taxa, the NISP of bearded seal, *Erignathus barbatus,* ringed seal, *Pusa hispida,* harp seal, *Pagophilus groenlandicus* and hooded seal, *Cystophora cristata,* will also be reported (see Section 2.1 and Section 3.3 for further details).

***2.5.4. Artefacts.*** For each assemblage, evidence of walrus artefacts will be recorded as follows: artefact type as reported (e.g. harpoon head), artefact skeletal element (tusk, bone, hide, cheek tooth), and the number of artefacts of this type.

### 2.6 Biases and quality assessment

Various factors can affect the accuracy and precision of zooarchaeological data, such as the excavation method employed, site preservation conditions, butchery practices, dating methods, identification methods, and the level of detail in georeference reporting, among other factors ^60, 122– 124^. An additional caveat when aiming to draw conclusions regarding the distribution of faunal remains is the likelihood of trade. Animal commodities such as tusk ivory can be found far afield from original collection or hunting sites. For example, intercontinental trade of tusks has been demonstrated as early as the 12th century CE ^61^, and long-range exchange within and between regions can be inferred at a variety of dates ^125^. The distribution of traded walrus objects is an important proxy for the scale of demand on walrus populations (see 22), but must be differentiated from evidence of local walrus hunting. Association between the geodistribution of skeletal faunal remains (excluding tusks) and artefacts (including tusk ivory) will be used to identify areas where local resource utilisation was unlikely (areas with artefacts but not other faunal remains).

Many biases are challenging to assess in a comparable way using published evidence, but where practicable the contribution of key biases will be evaluated using quality criteria on an ordinal scale (1 - 3; weak, medium, strong; 0 - data recorded but will not typically be used in analysis). This information will be used to subset data into bins of lower and higher quality and conduct sensitivity analyses.

***2.6.1 Dating.*** Dates associated with archaeological assemblages alongside the method used to assess chronology will be accepted as reported in the published literature. Chronological reliability will be assessed according to the criteria established in 107 as follows:

**A. Chronological timespan**

1. Assemblages spanning more than 500 years will be classified as temporally uninformative (0).
2. Those with a chronological range between 301 and 500 years will be classified as data quality (1).
3. Those with a chronological range of between 201 and 300 years will be classified as data quality (2).
4. Those with a chronological range of 200 years or less will be classified as data quality (3).

**B. Chronological method**

1. Assemblages lacking a reported date will be classified as temporally uninformative (0).
2. Assemblages with an estimated date, where the dating method used is not clearly specified, or, where dating is based on radiocarbon assays that lack sufficient information for recalibration using current best practice (including marine reservoir correction where relevant), will be classified as data quality (1).
3. Sites (and their associated assemblages) dated through typology, stratigraphy and/or chronometric methods, but lacking quantified and up-to-date estimates of error, will be classified as data quality (2).
4. Sites using chronometric methods and providing primary data (e.g. radiocarbon dates that can be recalibrated) will be classified as data quality (3). In instances where uncalibrated radiocarbon dates are available, recalibration including marine reservoir correction (where relevant) will be performed using best practices during downstream analysis.

***2.6.2. Taxonomic identification.*** Fauna from archaeological sites are frequently fragmented, and certain osteological features crucial for definitively identifying species may be absent ^121^. In these instances, walrus bone specimens may be reported as “large unidentified pinniped” or “pinniped” without any further taxonomic information (e.g. 126). Advances in biogeochemical and biomolecular techniques including genetic and proteomic analyses have made it possible to assign fragmented faunal remains to species, although only in recent publications ^127, 128^. Because of these limitations, taxonomic reliability will be assessed according to the criteria established in 107 as follows:

1. Faunal identifications labelled ‘Unidentified pinniped’ will be classified as taxonomically uninformative (0) and only reported if studies also contain taxonomically-resolved faunal data (see Section 2.4).
2. Fauna labelled as ‘Unidentified large pinniped’ or equivalent will be classified as data quality (1), as depending on distribution this may also contain other large pinnipeds (e.g. Northern Elephant seal, *Mirounga angustirostris*).
3. Fauna of greater taxonomic resolution (e.g. *walrus, Odobenus*, *Odobenus rosmarus*) based on general zooarchaeological assessments alone will be classified as data quality (2).
4. Faunal identifications achieved through ZooMS, aDNA or specific morphological criteria will be classified as data quality (3).

***2.6.3. Location.*** As discussed in 107, zooarchaeological NISP data are often associated with spatial information of varying degrees of precision. For example, the precise location of an archaeological assemblage is not always available, or can only be obtained from low resolution maps within site reports. In such cases, and when precise locations are not legally protected, supplementary information or cross-referencing with tools like Google Maps (or similar applications) will be used to determine the exact location of the site and/or to enhance precision. Quality will be assessed as follows:

1. Faunal remains without location data will be classified as spatially uninformative (0) and only reported if studies also contain spatially-resolved faunal data (see Section 2.4).
2. Faunal remains affiliated with a broad spatial scale (e.g. country, province, state, county) will be classified as data quality (1).
3. Faunal remains without a site-specific georeference, but that can be pinpointed within 1 degree of latitude and longitude, or where specific site coordinates are provided but at a resolution of more than 1 degree latitude and longitude, will be classified as data quality (2).
4. Faunal remains affiliated with specific site coordinates with precision below 1.0 degree latitude and longitude will be classified as data quality (3).

***2.6.4 Local resource utilisation/hunting events versus trade.*** A correlation between the distribution of skeletal faunal remains (excluding tusks) and artefacts will be used to identify areas where local resource utilisation was likely versus unlikely as follows:

1. Tusks or artefacts that are known to be commonly traded goods that fall outside of the spatial range of skeletal elements (excluding teeth and tusks) will be classified as data quality (0).
2. Tusks or artefacts that are known to be commonly traded goods that fall within the spatial range of skeletal elements (excluding teeth and tusks) will be classified as data quality (1).
3. Walrus NISP (bone) that are geolocated but not associated with a known hunting site or event will be classified as data quality (2).
4. Walrus NISP (bone) that are geolocated and can be associated with a hunting site or event will be classified as data quality (3).

## 3. Analytical approach

Using the resulting dataset, we propose to perform three analyses (noting that although we plan to incorporate the following approaches, this will be dependent on the characteristics of the ultimate dataset, and thereby subject to change) to address the following three key research aims:

1. To record when and where there is evidence of human societies utilising walruses over the past two millennia.
2. To identify areas of accelerated extractions across the discontinuous circumpolar Arctic distribution of walruses and if accelerated extractions are identified, to explore the relationship between increased resource extractions and societal and/or technological changes in hunting strategies, and/or changes in demand for walrus products.
3. To identify the environmental correlates associated with spatiotemporal variation of walrus faunal remains.

### 3.1. Spatiotemporal variation in walrus resource utilisation over the past two millennia

To investigate temporal variation in the resource use of walrus between 1 CE - 2000 CE on a global scale, aoristic sum analysis will be used on the aggregated NISP data following ^124^. Aoristic sum analysis will be repeated using a variety of discrete time bins (100, 200 and 300 yr time intervals) and the associated R Package archSeries. To identify spatiotemporal variation in zooarchaeological walrus data three statistical approaches will be used. First, aoristic sum analysis will be repeated as above but using NISP data aggregated at two spatial hierarchical levels: ocean-basin only; country nested within ocean-basin. Second, walrus faunal counts (NISP) will be binned into one of six time periods (1 CE - 299 CE; 300CE-599CE; 600CE-999CE; 1000CE-1299CE; 1300CE - 1599CE; 1600 CE - 1999 CE) and aggregated into hexbins of 1 degree × 1 degree decimal longitude and latitude. When associated chronology spans two time bins, the chronological midpoint will be used to assign time bins. Spatial hexbins will then be converted to either walrus present or walrus absent. Spatial variation in the occurrence of walrus over time (between 1 CE and 2000 CE) will then be assessed using Bayesian Additive Regression Trees (BARTs) and fuzzy logic following similar methods to 129.

We expect artefacts made from walrus remains to cover a larger spatial area and at higher abundances inland relative to skeletal elements because these items are more likely to travel further from hunting sites through trade networks (e.g. 49). To assess time-space instances when artefacts were subject to higher rates of trade relative to ecofacts (“unworked” walrus bone/tusk), walrus faunal counts (NISP) or counts of artefacts will be spatiotemporally aggregated into hexbins as previously described for NISP but converted to relative abundances (instead of presence/absence) across the spatiotemporal dataset (scaled between 0 - 1) independently for artefacts and ecofacts (NISP). Using these datasets, a spatial correlation will be performed using the modified t-test and the r package SpatialPack ^130^. This will be used to identify whether the locations of artefacts differ from ecofacts. If a difference is identified, generalized linear models will be used to identify whether artefacts are found further inland (distance from the coast) and more southerly (latitude) relative to ecofacts.

### 3.2. Accelerated extractions and societal/technological changes in walrus hunting strategies AND accelerated occurrence of trade (artefactual evidence)

From the aoristic sum analysis described in 3.1 (country, ocean-basin, or global), acceleration in walrus resource utilisation (accelerated extractions) will be statistically identified by measuring the average rate of change per 100-year time bin as follows:


$$m_{t}=\frac{\Delta y_{t}}{\Delta x_{t}}$$

Whereby *m _t_* is the average rate of change between two time points, *y* is faunal counts and *x* is time. The rate of change across the time period (1 CE - 2000 CE) will then be assessed for homogeneity, with a homogeneous line representing a constant rate of change and therefore no acceleration or deceleration.

Where practicable, to infer whether accelerated extractions (if any) are associated with changes in human population size, cross-correlations between faunal counts and cumulative radiocarbon dates (a proxy for human population size, see 131– 133) will be conducted using the R Package *tseries.* Moreover, to understand whether acceleration events are associated with cultural and/or technological changes, piecewise linear regressions will be used to compare faunal counts with the timing of known transformations in the methods of walrus hunting; for example, the introduction of firearms that made large walruses much easier to hunt from afar and therefore less risky to humans ^134^. Cumulative radiocarbon dates and climatic variables will be included as covariates. As NISP data are associated with a chronological range rather than fixed values, the outputs of the aoristic sum analysis will be subsampled prior to piece-wise linear regressions, repeated 100 times per locality, and results compared.

### 3.3. Spatiotemporal variation in Arctic and subarctic environments using proxies of environmental change

To infer historical environmental drivers of walrus distribution, the probability of occurrence of walrus (aggregated into 1 degree x 1 degree hexbins as above) within each time period will be modelled using Bayesian Additive Regression Trees (BARTs) and a set of environmental covariates (harp seal presence; bearded seal presence; ringed seal presence; hooded seal presence; mean annual sea surface temperatures; annual variability in SST; isothermality; soil pH in H20; clay content in mass fraction (CLYPPT); distance to coast). BART models incorporate presence and absence data using a logit link structure as follows:


$$P(Y)=f(x)=\sum_{j=1}^{m}g(x;T_{m};M_{m})+\epsilon$$

Where Y is a vector of the response variable (counts of walrus bones) for each grid cell and P represents the probability of walrus occurrence for each grid cell given the data and the model predictors, *g(x)*. *m* represents the distinct regression trees each composed of a tree structure T as an ensemble (T _1_, M _1_),..., (T _m_, M _m_) given each prediction *g(x)* of a set of trees. As faunal data will be associated with a chronological range rather than a fixed time period, one model will be constructed using the chronological midpoint of the associated assemblage and a second model will be constructed by randomly assigning assemblage data to per century time bins, i.e. NISP data associated with the following date range, 2nd century CE - 5th century CE, will be randomly assigned to either 2nd century CE, 3rd century CE, 4th century CE, or 5th century CE for a given model. Models will be repeated 100 times and random draws from model posteriors will be combined from all 100 models to produce the ‘mean’ model also referred to as the joint posterior distribution.

## 4 Outputs

Arctic and subarctic environments are currently undergoing rapid change ^65, 71^ and a good understanding of how walruses have historically responded to environmental change is essential in order to predict how they may respond to ongoing climatic changes. The collation and analysis of zooarchaeological data of walrus over the past two millennia will: (i) help to determine how walrus resource utilisation varied between 1 CE - 2000 CE; (ii) infer when accelerated extractions occurred and hypothesise how these might be related to cultural and technological changes in walrus hunting; and/or (iii) environmental change. Additionally, these data will provide insight into how past hunting events may have led to localised extirpations of walrus (e.g. Canadian Maritimes ^11^). These data will contribute to an open-access global atlas of historical marine exploitation.

## Data Availability

No data are associated with this article. Supplementary data tables including the PRISMA (preferred reporting items for systematic review and meta-analysis protocols) checklist can be downloaded from Zenodo ^135^ at: https://doi.org/10.5281/zenodo.10884775. Data are available under the terms of the Creative Commons Attribution 4.0 International license (CC-BY 4.0).
