# Supplementary material for: Archaeological evidence of resource utilisation of walrus, Odobenus rosmarus, over the past two millennia: A systematic review protocol
Source: Open Res Eur. 2024 Apr 24;4:86. [Version 1] doi: 10.12688/openreseurope.17197.1 (PMC11283631; doi:10.12688/openreseurope.17197.1)
Supplement: Peer review history

# Reviewer report: Reviewer response for version 1

**DOI:** 10.21956/openreseurope.18585.r42111

**Published:** 2024-07-27

**Authors:** Jeff Higdon

**Affiliations:** 1 Higdon Wildlife Consulting, Winnipeg, Canada

Buss et al. describe a protocol for systematically reviewing archaeological evidence for human use of walrus ( *Odobenus romarus* ) between 1 CE and 2000 CE. The authors will collate zooarchaeological evidence of walrus artefacts, tusks and bones across the Northern Hemisphere, primarily from English-language peer-reviewed indexing. The collated data will be openly available for the scientific community in three datasets.

 The authors have indexed their protocol to ensure reproducibility in the future, and the protocol is well-designed and comprehensive. The background material presents a detailed overview of the importance of walruses to Arctic ecology and human socio-economics and the history of walrus exploitation. The objectives are clearly stated and the reasoning behind them are sound. The methods to be used in the systematic review protocol are thoroughly described, from the initial literature screening to the data analyses and reporting. The proposed analyses techniques are well-suited for the study objectives and should offer useful information on historical walrus utilization in the context of cultural and environmental change.

 I have one suggestion:

 Faunal counts of four ice-obligate pinniped species - the authors will compile zooarchaeological NISP data of four ice-obligate seal species (bearded, ringed, harp, and hooded seal) that are found within the sources used for walrus data extraction (i.e., no independent searches). Data on these four species will be useful to the authors' goal of inferring environmental change over time, but I would encourage them to include harbour seal ( *Phoca hispida* ) data if encountered during the literature review as well. While not an ice-obligate species, changes in the frequency of harbour seals in zooarchaeological assemblages will provide complimentary evidence for environmental shifts (e.g., decreases in harp seal remains and increases in harbour seal remains) and could be included in Bayesian Additive Regression Trees (BARTs).

Is the study design appropriate for the research question?

Yes

Is the rationale for, and objectives of, the study clearly described?

Yes

Are sufficient details of the methods provided to allow replication by others?

Yes

Are the datasets clearly presented in a useable and accessible format?

Yes

Reviewer Expertise:

Biology, ecology and management of Atlantic walruses. Catch history reconstruction using a wide variety of source material including archaeological and historical data. History of Arctic wildlife species exploitation from pre-historic to modern times.

I confirm that I have read this submission and believe that I have an appropriate level of expertise to confirm that it is of an acceptable scientific standard.

# Reviewer report: Reviewer response for version 1

**DOI:** 10.21956/openreseurope.18585.r41070

**Published:** 2024-06-18

**Authors:** Aikaterini Glykou

**Affiliations:** 1 Stockholm University, Stockholm, Sweden

The paper “Archaeological evidence of resource utilisation of the great whales over the past two millennia: A systematic review protocol” by Danielle L. Buss et al. outlines a protocol for systematically reviewing archaeological evidence of human utilization of walrus ( *Odobenus rosmarus*) over the past two millennia. The goal is to collate data on walrus artefacts, tusks, and bones documented in the literature to understand the spatiotemporal distribution of walrus hunting and its societal impacts.

 The paper consists of four sections: 1. Background, 2. Methods, 3. Analytical Approach, and 4. Outputs. The systematic review protocol is well-designed with clearly stated rationale and objectives. The methods and statistical analyses are comprehensively detailed, ensuring the study's reproducibility and robustness. The anticipated conclusions are expected to be well-supported by the thorough and methodical approach outlined.

 The “Background” section provides an overview of the ecological and cultural significance of the walrus in Arctic and subarctic regions. It highlights the historical context of walrus hunting, especially the intensification due to European demand for ivory, and discusses the impact on walrus populations. The importance of walruses to Indigenous communities and their role in local ecosystems is also emphasized. The rationale and objectives are clearly stated and well-justified. The study aims to fill a gap in understanding the historical usage and distribution of walrus resources, which is essential for comprehending past ecological and societal dynamics.

 The next section, “Methods,” details how the systematic review protocol is designed to collate and analyze data on walrus remains and artifacts from archaeological sites. It describes the criteria for including studies, the predefined search terms, and the databases to be used (e.g., Web of Science, Scopus, JSTOR). It outlines the process for data extraction, including the types of data to be collected (e.g., faunal counts, artifacts) and the quality assessment measures to ensure the reliability of the data. Biases and quality assessment measures are also discussed, ensuring transparency and reproducibility.

 The “Analytical Approach” section explains how the collected data will be analyzed. It outlines the planned statistical analyses to identify spatiotemporal patterns in walrus exploitation, assess the impact of environmental and societal changes, and understand the historical distribution and utilization of walrus populations. This includes using Bayesian Additive Regression Trees (BARTs) and other statistical tools to model the probability of walrus occurrence and the relationship between human activities and walrus populations. The statistical techniques proposed are well-suited for the study's aims and appear to be capable of providing meaningful insights into the historical utilization of walruses. The use of BARTs for modeling environmental correlates is particularly relevant for understanding historical environmental changes.

 Finally, the “Outputs” section describes the expected results and their implications. It aims to provide insights into how walrus resource utilization varied between 1 CE and 2000 CE, identify areas of accelerated extractions, and hypothesize how these might be related to cultural and technological changes in walrus hunting or environmental changes. The data will contribute to an open-access global atlas of historical marine exploitation, helping to understand past human impacts on walrus populations and predict future responses to ongoing climatic changes.

 Some minor issues to consider:

1. Manual Data Entry:

Manual data entry into an OpenOffice database is time-consuming and prone to human error. Is there a more sophisticated option for automatic data extraction? Moreover, is OpenOffice robust enough to manage such large datasets while ensuring data integrity? The authors might consider other database systems that offer better scalability and data management features.

1. Interoperability:

It is unclear if the protocol supports interoperability with other databases and software tools. Clarifications or guidelines for data access and sharing could improve usability for future research. Ensuring that the data can be easily integrated with other systems will enhance the protocol’s utility and accessibility.

1. Exclusion of Google Scholar’s non-English results:

The exclusion of Google Scholar's non-English search results could lead to missing some relevant studies. While the rationale is understandable, have the authors considered incorporating machine learning tools to assist with the initial screening of large datasets to enhance efficiency? These tools could help manage the large volume of search results more effectively and identify relevant studies in multiple languages.

 The protocol presented by Danielle L. Buss et al. is comprehensive and methodologically sound. Addressing the above concerns could further enhance the protocol's efficiency, reliability, and applicability in future research.

Is the study design appropriate for the research question?

Yes

Is the rationale for, and objectives of, the study clearly described?

Yes

Are sufficient details of the methods provided to allow replication by others?

Yes

Are the datasets clearly presented in a useable and accessible format?

Yes

Reviewer Expertise:

Archaeolozoology, Bioarchaeology, Statistics

I confirm that I have read this submission and believe that I have an appropriate level of expertise to confirm that it is of an acceptable scientific standard.
